# The Effect of Dietary Quercetin on the Glutathione Redox System and Small Intestinal Functionality of Weaned Piglets

**DOI:** 10.3390/antiox8080312

**Published:** 2019-08-16

**Authors:** Jeroen Degroote, Hans Vergauwen, Noémie Van Noten, Wei Wang, Stefaan De Smet, Chris Van Ginneken, Joris Michiels

**Affiliations:** 1 Laboratory of Animal Nutrition and Animal Product Quality (LANUPRO), Department of Animal Sciences and Aquatic Ecology, Ghent University, Coupure Links 653, 9000 Ghent, Belgium; 2 Laboratory of Applied Veterinary Morphology, Department of Veterinary Sciences, University of Antwerp, Universiteitsplein 1, 2610 Wilrijk, Belgium

**Keywords:** weaned pigs, quercetin, glutathione, redox status, small intestine

## Abstract

Quercetin has been shown to alleviate mucosal damage and modulate the glutathione (GSH) redox system in the colon of rodents. In the current study, we assessed whether quercetin was able to mitigate small intestinal dysfunction in weaned pigs. Here, 224 weaned piglets were fed a diet containing quercetin at either 0, 100, 300, or 900 mg/kg diet until d14 post-weaning, followed by a common basal diet until d42. Eight animals per treatment were sampled at d5 and d14 post-weaning. In these animals, the small intestinal histomorphology, barrier function, and protein abundance of occludin, caspase-3, and proliferating cell nuclear antigen were assessed. None of these parameters were affected, and neither did quercetin improve performance up to d42 post-weaning. The GSH redox system was evaluated in blood, small intestinal mucosa, and liver. Quercetin did not affect the glutathione peroxidase, glutathione reductase, and glutamate–cysteine ligase activity in these tissues. In contrast, the hepatic glutathione transferase (GST) activity was significantly increased by quercetin supplementation at d5 post-weaning of 100, 300, and 900 mg/kg. Importantly, d5 was characterized by a more oxidized GSH redox status. To conclude, dietary quercetin had little effect on the small intestine, but did upregulate hepatic GST in the occurrence of redox disturbance.

## 1. Introduction

Quercetin, as the foremost representative of flavonols, has been extensively investigated for its beneficial effects on health [1]. With regard to the gastrointestinal tract, quercetin received a lot of attention in treating chronic intestinal inflammation. Different mechanisms, including protection against oxidative stress, preservation of epithelial barrier function, and immunomodulatory properties in the gut, are believed to be involved [2,3]. These biological effects could be valuable in weaned piglets, since these animals suffer from a disturbed gastrointestinal functionality during the first two weeks after weaning. Villus atrophy—mucosal barrier dysfunction in the small intestine—co-occurs with a state of acute inflammation and immune stimulation [4,5,6]. Therapy in weaned piglets mainly relies on the use of zinc and copper beyond nutritional requirements and the excessive use of antimicrobials. This raises major concerns for antimicrobial resistance [7]. In the quest for sustainable alternatives, quercetin prompted interest since this molecule showed excellent assets in an intestinal porcine epithelial cell (IPEC-J2) line [8]. Among the six antioxidants tested here, quercetin was most effective in reducing ROS and supporting barrier function. Growing evidence further indicates that quercetin is also a potent modulator of the glutathione (GSH) redox system. Glutathione transferase (GST) and glutamate-cysteine ligase (GCL) are the two principal enzymes upregulated by quercetin through interference with redox signaling mechanisms [9,10,11,12,13]. While GST enables the conjugation of GSH to proteins and xenobiotics, GCL catalyzes the first and rate-limiting step in the synthesis of GSH. The effects on other GSH-related enzymes, such as glutathione peroxidase (GPx) and glutathione reductase (GR), which respectively catalyze the oxidation of GSH and reduction of glutathione disulphide (GSSG), are not well documented. This though could be important since redox mechanisms are involved in tight junction regulation [14,15,16] and cell cycling from proliferation to apoptosis [17,18], and redox disturbance occurs during the weaning transition of piglets [19,20].

Research on the beneficial effects of quercetin in pigs is however limited [21,22] compared to the considerable amount of data generated in rodent models for experimentally induced colitis. In these experiments, quercetin and its naturally occurring glycosides quercetrin and rutin all demonstrated a remarkable potential to attenuate colonic mucosal damage [23,24,25,26,27,28,29,30,31,32,33,34]. When targeting the small intestine, quercetin could be the preferential source, as the beneficial effects are primarily ascribed to quercetin and not its metabolites [35]. Besides, its glycosides need an additional step of hydrolysis, either by lactase-phlorizin hydrolase at the brush border surface, by the intestinal microbiota, or intracellularly after active transport of monoglycosides [23,36,37]. This might not favor its functionality at the level of the small intestine.

After oral ingestion, quercetin is easily absorbed and further metabolized by the small intestine. Using an in situ perfusion model of the rat jejunum plus ileum, Crespy et al. (1999) found that two-thirds of the quercetin disappeared from the lumen. A high proportion of quercetin was also found to be re-excreted into the lumen as conjugated derivates [38]. Conjugation of orally administered quercetin with glucuronic and sulfuric acid appears to preferentially occur in the intestinal wall of pigs, which further explains the low apparent bioavailability of free, unchanged quercetin [37,39]. A recent study in rats demonstrated that the intestinal epithelium is the major site of glucuronidation, while the liver rather promotes sulfation and methylation of quercetin. Quercetin can be easily methylated by catechol-O-methyl transferase. However, this rate appears to be low in the pig [30,37,40]. The resulting glucurono- and sulfo-conjugated metabolites of quercetin and its methylated metabolites isorhamnetin and tamarixetin are the main circulating forms in plasma [37,40]. Importantly, glucurono-, but not sulfo-conjugates, can be hydrolyzed at the vascular level, yielding the aglycone, which can accumulate in tissues [36]. Accumulation is considered to be limited in pigs, as the tissue distribution of quercetin and its metabolites is not different between a single treatment and long-term dietary intake of quercetin [40]. Predominantly organs involved in the metabolism and excretion of quercetin, including the liver, small intestine and kidney, were observed to hold higher flavonol concentrations than plasma. Yet colon tissue contained three times the amount of jejunal tissue, and this almost exclusively as the quercetin aglycone [40]. The colon thus remains the main site for quercetin accumulation, although the small intestine extensively absorbs and metabolizes quercetin. As the concentration of the molecule dictates its potential effect, it thus remains unclear whether the small intestine would benefit from quercetin supplementation. Finally, it should be emphasized that the pharmacokinetics of quercetin and its glycosides is species-dependent [40,41]. In this study, we therefore aimed to elaborate on the effects of quercetin on the small intestinal mucosa in piglets during the weaning transition. We investigated the small intestinal functionality and the GSH redox at d5 and d14 post-weaning, respectively allowing to assess the prevention and the recovery of intestinal damage upon weaning.

## 2. Materials and Methods

### 2.1. Experimental Animals and Feeds

The experiment was carried out according to the guidelines of the Ethical Committee of the Faculty of Veterinary Sciences, Ghent University (Ghent, Belgium) for the humane care and use of animals in research. For this experiment, 224 piglets (Piétrain × Topigs hybrid) were selected at weaning from 32 litters at one farm. 119 Barrows and 106 gilts (4.0–8.5 kg) were weaned at 21 days of age. Selected piglets were inspected for health prior to transport to the trial facilities. Piglets were allocated to 32 pens, each holding seven piglets per pen, according to the protocol described below. Pens had a surface area of 2.10 m^2^/pen, and were equipped with full slatted floors, a single-sided dry feeder (60 cm wide) and one bowl drinker. From d0 to d14 post-weaning, piglets were fed the four different experimental weaner diets, varying in the dose of quercetin. The starter diet was then offered from d14 to d42 post-weaning and was identical for all treatments. Diets were barley-, wheat- and soy-based meals, formulated to meet or exceed recommendations by the Dutch Centraal Veevoederbureau (1997) [42]. The weaner and starter diets contained basal levels of vitamin E (54 IE/kg), butylated hydroxytoluene (75 mg/kg) and ethoxyquin (38 mg/kg), but no in-feed antibiotics or pharmacological doses of zinc oxide (supplemental Zn^2+^: 100 mg/kg). More details on the ingredient and nutrient composition can be found in Table 1.

### 2.2. Experimental Design and Measurements 

The experiment included four dietary treatments, i.e., the weaner diet (d0-14 post-weaning) contained 0 (QUE0), 100 (QUE100), 300 (QUE300) or 900 (QUE900) mg quercetin per kg diet. The diets were prepared by first preparing one batch of basal weaner diet, dividing this in four equal batches, and mixing the appropriate amount of quercetin into the diet. Quercetin (CAS 117-39-5) was obtained from Sigma Aldrich (Overijse, Belgium) as a ≥ 95% grade powder. Treatments were replicated in eight pens with seven animals per pen. First, piglets were allocated to the pens in order to stratify for mean body weight and litter origin. Here, eight littermate piglets were divided over eight neighboring pens, and seven different litters were used to reach a total of seven piglets per pen. This process was repeated four times, resulting in a randomized block design where each treatment was randomly assigned to two pens per block. At minimum, two out of seven piglets per pen represented each gender. Piglets were weighed individually at d0, d5, d14, d28, and d42 post-weaning. Feed intake was registered at the pen level by weighing feeders and residual feed. Average daily gain (ADG), average daily feed intake (ADFI), and feed conversion ratio (FCR) were calculated for following intervals; d0–5, d5–14, d14–28, d28–42, and d0–42 post-weaning.

An ordinal scoring system was used by a trained individual to daily score the fecal consistency of each pen throughout the 14d post-weaning period, and included three categories: 1 = hard or slightly moist feces, clearly formed, normal; 2 = moist or soft feces, but still with a definite form, sticky; and 3 = watery or liquid feces, unformed, diarrhea. Diarrhea incidence was simultaneously assessed by counting pigs with a fecal consistency score of 3, including clear signs of diarrhea: filthy, wet backside and tail, dehydrated, loss of condition, and irritation of the skin around the anus. Antibiotic treatments were registered and were limited to individual intramuscular injections.

### 2.3. Sample Collection

Both at d5 and d14, one piglet per pen was euthanized and sampled. Here, the animal having the body weight closest to the average body weight of the pen at that time was selected, irrespective of their gender. No fasting period was included. Animals were anaesthetized by electrical stunning and during exsanguination, blood was collected in heparinized tubes containing 200 µL bathophenanthroline disulfonic acid and K3-EDTA tubes. After immediate centrifugation (3000 × *g*, 10 min), K3-EDTA plasma was harvested and stored at −20 °C for the analysis of malondialdehyde (MDA), GPx, GR, GST, and GCL, while heparinized plasma was discarded, and erythrocytes were lysed with metaphosphoric acid and intense vortexing. Samples were then centrifuged (3000 × *g*, 10 min), and 0.5 mL of the resulting acid extract was supplemented with γ-glutamyl-glutamate, snap frozen and stored at −80 °C. Meanwhile, a first 10 cm section was obtained at 75% (distal jejunum) of the total small intestinal length and was used in the Ussing chamber assay to determine the macromolecular permeability according to the principles describe below. A second section of intestinal tissue (20 cm), both at 5% (duodenum) and 75%, was harvested for its mucosa by scraping the mucosal surface with a glass slide. A first mucosal subsample was homogenized in ice cold perchloric acid (1:10 m:v; 1.16 M) with a Braun Potter homogenizer (1000 rpm, 30 s). Homogenates were centrifuged (10,000 × *g*, 15 min, 4 °C), after which an aliquot was supplemented with γ-glutamyl-glutamate, snap frozen in liquid nitrogen and stored at −80 °C. These extracts were used for the quantification of GSH and GSSG. At the same time, a second mucosal subsample was homogenized in a 1% triton-X-100 phosphate-buffer (pH 7.0, 50 mM, 1:10 m:v), and was centrifuged (10,000 × *g*, 15 min, 4 °C). The supernatants were snap frozen and stored at −20 °C pending the analysis of the total protein content, MDA, GPx, GR, GST, and GCL. A third mucosal subsample was snap frozen and stored at −80 °C awaiting the analysis of protein abundance. A last section of intestinal tissue (5 cm) at both intestinal sites was placed in 4% paraformaldehyde solution (pH 7.4) for two hours, followed by rinsing with phosphate-buffered saline and further processed according to standard procedures for histomorphology. Finally, also the liver was excised and perchloric acid and phosphate-buffered extracts were made and stored identical to the procedures for small intestinal mucosa. 

### 2.4. Laboratory Analysis

The GPx activity assay, as described by Hernandez et al. (2004) [43], was based on measuring the consumption of NADPH at 340 nm, in a reaction mixture containing NADPH, GR and GSH. One unit of GPx activity was equivalent to one µmol NADP+ from NADPH per minute at pH 8.0 and 25 °C. The activity of GR was determined according to the method of Carlberg and Mannervik (1985) [44]. This was based on the reduction of GSSG by NADPH in the presence of GR and monitoring the disappearance of NADPH at 340 nm. One unit of GR caused the oxidation of one μmole of NADPH at 25 °C (pH = 7.5). The GST activity was determined by measuring the increase in absorbance at 340 nm, associated with the conjugation of GSH with 1-chloro-2,4-dinitrobenzene (CDNB) catalyzed by GST [45]. One unit of GST conjugated one µmol of CDNB with GSH per minute at 25 °C (pH = 6.5). The activity of GCL was determined according to the method of White et al. (2003), based on the reaction of naphthalene-2,3-dicarboxialdehyde (NDA) with the γ-glutamylcysteine (γ-GC) produced in a medium containing ATP, glutamic acid, cysteine and sample (1725) [46]. Fluorescence intensity was measured at 472 nm excitation and 528 nm emission. One unit of GCL was defined as one nmol of NDA-γ-GC formed per minute at 25 °C (pH = 7.5). The thiobarbituric acid reactive substances method was employed to quantify MDA as a marker for lipid peroxidation [47]. Finally, a bicinchonic acid assay was used to quantify the protein content in phosphate-buffered mucosal and hepatic extracts [48].

The concentrations of GSH and GSSG were determined by the methodology of Yoshida et al. (1996) [49], relative to γ-glutamyl-glutamate as internal standard and to GSH and GSSG external standard solutions. The analytical procedure was identical as in earlier research [50,51]. In brief, in involves the quantification of N-(2,4-dinitrophenyl) derivates by reversed-phase HPLC and with UV absorption measurement at 365 nm. The GSH/GSSG E_h_ values (in mV) were calculated by using the appropriate forms of the Nernst equation, at pH 7.4 and 37 °C, whereby concentrations of GSH and GSSG were expressed in molarity [52]:

$$GSH/GSSG\;E_{h}=-264-\;\frac{61.5}{2}\;\times \;log_{10}\left(\frac{GSH^{2}}{GSSG}\right)\;\left[mV\right]$$

The mucosal permeability for 4-kDa fluorescein isothiocyanate dextran (FD4; Sigma-Aldrich, Overijse, Belgium) was assessed in an Ussing chamber setup. Three distal jejunal segments per pig were striped from its serosal layers and mounted into inserts with an opening area of 1.07 cm^2^ within 15 min post-mortem. Further procedures were identical as earlier described [53]. Samples in the acceptor compartment were taken at 20-min intervals between 40 min and 100 min after mounting the segment, and the fluorescent signal was captured using excitation filter at λ = 494 nm and emission filter at λ = 521 nm. The change in concentration over time (dc/dt) was calculated from the slope of concentration-time curve (µg/mL per s). The apparent permeability coefficient (Papp) was then calculated following the equation described by Neirinckx et al. (2011) [53].

The standard histomorphology procedures included dehydration and paraffin embedding, whereafter 5 μm transverse sections were mounted on slides and stained with hematoxylin-eosin. Villus height, villus width and crypt depth were measured in 50 well-oriented villi and adjacent crypts using an Olympus BX61 microscope and image analysis software (analySIS Pro, Olympus, Aartselaar, Belgium), in at least 10 sections per tissue sample.

The protein level of occludin, caspase-3 and proliferating cell nuclear antigen (PCNA) were assessed using commercially available enzyme-linked immunosorbent assays (ELISA) (Cloud-Clone Corporation^®^, Houston, TX, USA), respectively with the product code SEC228Hu, SEA626Hu, SEA591Hu. Therefore, mucosal samples were crushed, dissolved in phosphate-buffered saline (pH 7.4, 10 mM), sonicated (6 times 5 min, 4 °C), kept on ice for 30 min, and centrifuged (10,000 × *g*, 2 min, 4 °C). The supernatant was diluted to a total protein concentration of 10 ng/µL. Samples were then processed on a sandwich ELISA plate and the experiment was performed according to the manufacturer’s instructions. Absorbance was measured at 450 nm and 25 °C, and values of protein abundance were expressed as fmol per mg protein.

### 2.5. Statistics

Performance and analytical data were subjected to a general linear model module in IBM SPSS Statistics version 24.0 (SPSS Inc., Chicago, IL, USA). First, data were checked for violations on the normality and homoscedasticity assumptions. The individual piglet was considered as the experimental unit for analytical variables (*n* = 8), and the model contained dietary treatment (QUE), day post-weaning (DAY) and their interaction (DAY × QUE) as fixed effects. Performance data at the pen level (*n* = 8) were analyzed per time point, and the model contained the fixed effect of dietary treatment and the random effect of block. Data are presented as least squares means with the standard error of the mean (SEM) in tables, or with the standard deviation (SD) in figures. Differences were considered significant at *p* ≤ 0.05, and statistical tendencies were assumed when 0.10 > *p* > 0.05. Tukey’s multiple comparison test was applied to discriminate the dietary treatments.

Principal component analysis (PCA) was used as a dimension reduction technique, to evaluate if treatments groups had altered overall GSH redox status and gut functionality. Data were standardized, i.e., for each observation, the value was subtracted with the grand mean and divided by the standard deviation. A first PCA was conducted with the data from 23 variables on the GSH redox status at d5 and d14 post-weaning, being the GSH and GSSG concentrations and the activities of GSH-related enzymes in erythrocytes or plasma, duodenal mucosa, distal jejunal mucosa, and liver. The GSH/GSSG E_h_ was not included since this variable is computed from the GSH and GSSG concentrations and resulted in multicollinearity. The plasma GR activity was not included since no detectable amounts of activity could be monitored. MDA concentrations were not included in order to attain a subject-to-variable ratio of approximately three to one. An initial PCA gave rise to seven principal components with an eigenvalue higher than one. The first two principal components, with initial eigenvalues of 4.3 and 3.2, were retained for a final PCA. A second PCA analysis contained all variables on histomorphology, FD4 flux and PCNA, caspase-3 and occludin protein abundance. The subject-to-variable ratio was approximately six to one. The first two principal components, with initial eigenvalues of 3.1 and 2.1, were retained from a list of three components with an eigenvalue higher than one. For both PCA’s, the Kaiser-Meyer-Olkin statistic of sampling adequacy was ≥ 0.6, and Bartlett’s test of sphericity indicated multivariate normal distribution with zero covariance (*p* < 0.001). Varimax rotation with Kaiser normalization was applied to ensured maximal sum of the variances of the squared loadings matrix. Piglets received component scores for the two principal components by using the least squares regression approach. An identical statistical model was used as for the analytical variables, to test the effect DAY, QUE and DAY × QUE on the scorings of those final components.

## 3. Results

### 3.1. Animal Performance

During the first 14 days of the experiment, when the four different experimental diets were fed, no significant differences were found on animal performance (Table 2). Piglets fed the QUE300 diet lost weight until d5 post-weaning, whereas other treatment groups displayed a slightly positive ADG in this period (*p* > 0.05). The ADFI at d0-5 and d5-14 post-weaning was also not found to differ between treatments, indicating that piglets had no preference nor aversion towards quercetin up to a dose of 900 mg/kg diet. Similarly, FCR was not affected by dietary treatment in this phase. At d14 post-weaning, piglets were switched from the weaner diet to the starter diet, which did not contain quercetin and thus was identical for all treatments. Remarkably, treatment effects were observed in this phase of the experiment. The BW at d28 and d42 post-weaning were significantly increased with respectively 0.96 and 1.39 kg more for treatment QUE900, as compared to QUE300. This was reflected in a significantly higher ADG for treatment QUE900 compared to QUE300 when considering the entire experiment (d0–42 post-weaning). Also, feed intake was influenced by the dietary treatment. From d28 to d42 post-weaning, the ADFI was increased with 9% in QUE900 when compared to the control (QUE0) (*p* = 0.046). Lastly, treatment did not affect the FCR at any given time, indicating that the increased growth in QUE900 was the result of a higher feed intake, rather than a more efficient feed utilization.

Diarrhea incidence and watery feces were most frequently observed between d5 and d12 post-weaning (data not shown). The average fecal consistency score was unaffected by dietary treatment (*p* > 0.05), and the overall score amounted to 1.80 on a scale from 1 (firm) to 3 (watery). Incidence of diarrhea was moderate, as on average 7.2%, 7.0%, 6.7% and 2.9% (*p* > 0.05) of the piglets showed clear visual indications of diarrhea, respectively for treatment QUE0, QUE100, QUE300 and QUE900. In total, four animals had to be removed from the study due to outlier performance, i.e., one animal from treatment QUE0, two from QUE100, and one from QUE900. These animals showed a reduced vitality, failed to thrive for several days, and did not respond to treatment with meloxicam. In total, only five antibiotic treatments with enrofloxacine were administered to piglets showing signs of arthritis, gastrointestinal or respiratory infections. This was unrelated to the dietary treatment. Furthermore, two piglets unexpectedly died during the experiment. One animal from treatment QUE100 was found dead on d2 post-weaning, and one animal from treatment QUE0 was found dead at d29 post-weaning. In both cases of sudden death, these animals were not found to be anorexic or lethargic. No post-mortem autopsy was executed.

### 3.2. Lipid Peroxidation in Different Tissues

Malondialdehyde was measured in different tissues as a marker for lipid peroxidation (Table 3). The results indicate that dietary quercetin did not influence the MDA concentrations in tissues (QUE: *p* > 0.05; DAY × QUE: *p* > 0.05). However, MDA levels were significantly affected by DAY in all measured tissues (*p* ≤ 0.05). Plasma levels were 17% higher on d5 versus d14 post-weaning, whereas duodenal and distal jejunal MDA was respectively 29% and 16% higher on d5 post-weaning. Finally, also hepatic MDA levels were higher at d5 when compared to d14 post-weaning (+25%).

### 3.3. Responses of the Glutathione Redox System in Different Tissues

#### 3.3.1. The Glutathione Redox System in Blood

**Figure 1:**
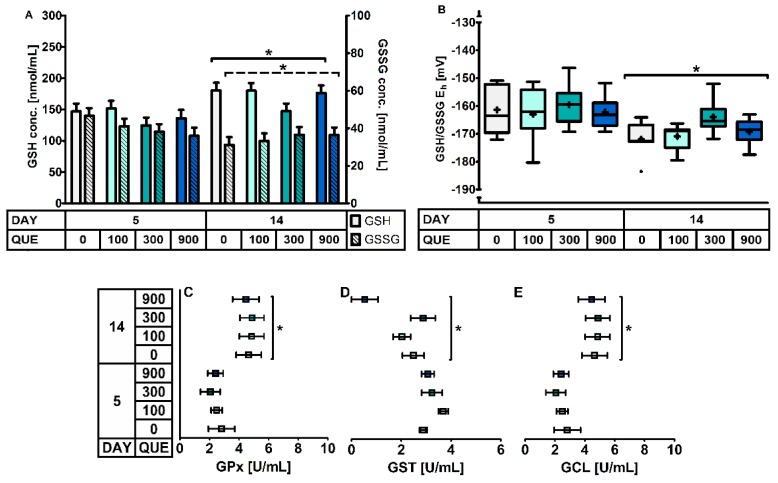
The glutathione redox system in blood of piglets at d5 and d14 post-weaning (DAY), fed a diet containing 0, 100, 300, or 900 mg quercetin/kg diet (QUE) during the first 14d post-weaning. Results are presented as least squares means with SD. Significance levels of main effects and interaction terms are presented in Table 4: (**A**) glutathione (GSH) and glutathione disulphide (GSSG) concentrations in erythrocytes; (**B**) Glutathione redox status (GSH/GSSG E_h_) in erythrocytes; (**C**) Glutathione peroxidase (GPx) activity in plasma; (**D**) Glutathione transferase (GST) activity in plasma; (**E**) Glutamate-cysteine ligase (GCL) activity in plasma. * Represents the effect of DAY across other factors (*p* ≤ 0.05).

Including quercetin in the diet did not impact on the glutathione redox system in erythrocytes or plasma (Table 4, Figure 1). Glutathione-related enzymes were not affected by QUE and DAY × QUE. The GSH levels in erythrocytes tended to be affected by QUE, with lower levels in treatment QUE300 (*p* = 0.077). Moreover, the GSH redox system responded differently at d5 versus d14 post-weaning irrespective of dietary treatment (*p* ≤ 0.05). Here, d5 post-weaning was characterized by 18% lower GSH levels, 18% increased GSSG levels, and consequently a more oxidized erythrocyte GSH/GSSG Eh (+7 mV). This was joined by a 26% higher GST activity and a 48% lower GCL activity in plasma on d5 post-weaning. Plasma GPx activity was also significantly lower (−19%) on d5 than d14 post-weaning. We could not confirm that this was combined with altered activity of GR, as our current method yielded values below the detection limit. 

#### 3.3.2. The Small Intestinal Mucosal Glutathione Redox System 

**Figure 2:**
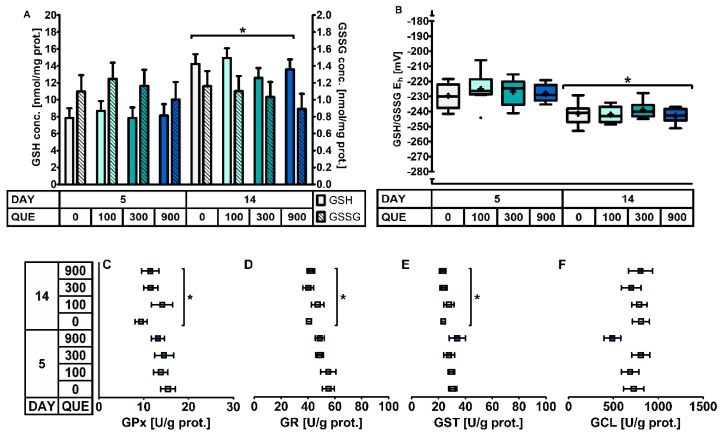
The glutathione redox system in the duodenal mucosa of piglets at d5 and d14 post-weaning (DAY), fed a diet containing 0, 100, 300, or 900 mg quercetin/kg diet (QUE) during the first 14d post-weaning. Results are presented as least squares means with SD. Significance levels of main effects and interaction terms are presented in Table 5: (**A**) glutathione (GSH) and glutathione disulphide (GSSG) concentrations; (**B**) Glutathione redox status (GSH/GSSG E_h_); (**C**) Glutathione peroxidase (GPx) activity; (**D**) Glutathione reductase (GR) activity; (**E**) Glutathione transferase (GST) activity; (**F**) Glutamate-cysteine ligase (GCL) activity. * Represents the effect of DAY across other factors (*p* ≤ 0.05).

The small intestinal GSH redox system was quantified both in the duodenal and distal jejunal mucosa. In the duodenal mucosa (Table 5, Figure 2), no effect of QUE nor DAY × QUE on the different components of the GSH redox system were observed (*p* > 0.05). However, similar as in plasma, the day post-weaning had an effect. The GSH/GSSG E_h_ was 14 mV higher on d5 versus d14 post-weaning. This was accompanied by a 40% lower GSH level (*p* < 0.001). GSSG concentrations did not to differ between d5 and d14 post-weaning. This drop in duodenal GSH was not associated with an altered GSH synthesis rate, as the GCL activity was similar at d5 and d14 post-weaning (*p* > 0.05). Instead, the drop in duodenal GSH was rather instigated by a 24% higher GST activity on d5 post-weaning. Piglets at d5 were also characterized by a higher GSH cycling redox activity, as both GPx (+22%) and GR (+22%) were significantly higher as compared to d14 post-weaning.

**Figure 3:**
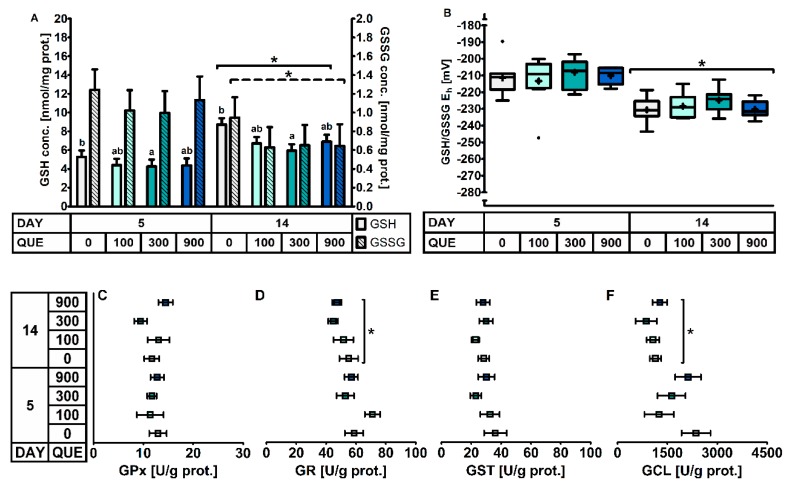
The glutathione redox system in the distal jejunal mucosa of piglets at d5 and d14 post-weaning (DAY), fed a diet containing 0, 100, 300, or 900 mg quercetin/kg diet (QUE) during the first 14d post-weaning. Results are presented as least squares means with SD. Significance levels of main effects and interaction terms are presented in Table 6: (**A**) glutathione (GSH) and glutathione disulphide (GSSG) concentrations; (**B**) Glutathione redox status (GSH/GSSG E_h_); (**C**) Glutathione peroxidase (GPx) activity; (**D**) Glutathione reductase (GR) activity; (**E**) Glutathione transferase (GST) activity; (**F**) Glutamate-cysteine ligase (GCL) activity. * Represents the effect of DAY across other factors (*p* ≤ 0.05).

In the distal jejunal mucosa (Table 6, Figure 3), QUE significantly affected the GSH concentration irrespective of the day post-weaning. Here, GSH levels were found to be decreased with 27% in treatment QUE300, as compared to QUE0. This decrease was not associated with any other change in the activity of GSH-related enzymes. The activity of GPx, GR, GST and GCL were unaffected by QUE or DAY × QUE (*p* > 0.05). Finally, although the GSSG levels were not different between dietary treatments, the altered GSH levels did translate in an altered GSH/GSSG E_h_. Similar to the duodenal mucosa, the distal jejunal GSH/GSSG E_h_ indicated a more oxidized GSH redox status on d5 versus d14 post-weaning (+18 mV: *p* < 0.001). This was caused by significantly lower GSH levels (−35%) and significantly higher GSSG levels (+53%) on d5 versus d14 post-weaning. Strangely, the activity of the different GSH related enzymes did not correlate with this observation, since the GCL and GR activity were found to be increased by respectively 69% and 20% on d5 versus d14 post-weaning, and GPx and GST activity were not affected by DAY (*p* > 0.05).

#### 3.3.3. The Hepatic Glutathione Redox System

**Figure 4:**
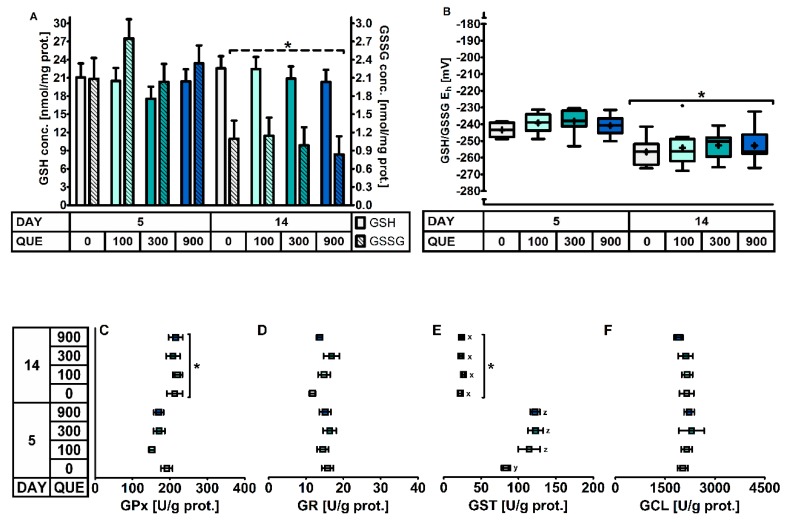
The glutathione redox system in the liver of piglets at d5 and d14 post-weaning (DAY), fed a diet containing either 0, 100, 300, or 900 mg quercetin/kg diet (QUE) during the first 14d post-weaning. Results are presented as least squares means with SD. Significance levels of main effects and interaction terms are presented in Table 7: (**A**) glutathione (GSH) and glutathione disulphide (GSSG) concentrations; (**B**) Glutathione redox status (GSH/GSSG E_h_); (**C**) Glutathione peroxidase (GPx) activity; (**D**) Glutathione reductase (GR) activity; (**E**) Glutathione transferase (GST) activity; (**F**) Glutamate-cysteine ligase (GCL) activity. * Represents the effect of DAY across other factors (*p* ≤ 0.05). ^x,y,z^ Represents the effect of QUE × DAY (*p* ≤ 0.05).

In the liver (Table 7, Figure 4), quercetin supplementation induced a higher GST activity on d5 post-weaning (DAY × QUE: *p* = 0.036), where GST activity increased with 37% in QUE100, 48% in QUE300 and 47% in QUE900, as compared to the control (QUE0). GST activity was not affected by QUE on d14 post-weaning (*p >* 0.05). On average, the GST activity was 78% lower on d14 versus d5 post-weaning (DAY: *p* < 0.01). No other effects of quercetin supplementation on the hepatic GSH redox system were observed (*p >* 0.05). Similar to blood and small intestinal mucosa, piglets on d5 post-weaning exhibited a more oxidized GSH redox status (+14 mV, *p* < 0.001) in the liver. This resulted from significantly higher hepatic GSSG levels (+126%) on d5 versus d14 post-weaning, since GSH levels were not different between those two days (*p* > 0.05). These observations were peculiarly linked with a decrease and not an increase in GPx activity on d5 post-weaning. The four-fold higher GST activity on d5 post-weaning, irrespective of dietary treatment, does however point towards a different GSH utilization, and thus a potential loss of hepatic GSH on d5 post-weaning. Finally, the activity of GR and GCL were not significantly affected by DAY. 

### 3.4. Small Intestinal Histomorphology and Barrier Function

Both in the duodenal and the distal jejunum, quercetin supplementation did not affect the histomorphological characteristics (Table 8). Villus length, villus width, crypt depth, and villus/crypt ratio were not altered by QUE (*p* > 0.05) or DAY × QUE (*p* > 0.05). Likewise, the protein abundance of PCNA and Caspase-3, measured in the distal jejunal mucosa, did not differ (QUE: *p* > 0.05; DAY × QUE: *p* > 0.05). These proteins are respectively involved in cell proliferation and apoptotic cell death, and thus indicate that quercetin did not alter cellular turnover in the jejunal mucosa. On the other hand, villus width in the distal jejunum increased with 9% on d14 versus d5 post-weaning (*p* ≤ 0.05). Also, crypts were on average 25% deeper on d14 compared to d5 post-weaning (DAY: *p* < 0.001), both in the duodenum and the distal jejunum. Although villus length did not differ between d5 and d14 post-weaning at both intestinal sites (*p* > 0.05), the difference in crypt depth resulted in approximately a 20% reduction in villus/crypt ratio on d14 versus d5 post-weaning (*p* ≤ 0.05).

The FD4 flux across the distal jejunal mucosa, as a marker for epithelial paracellular transport, was not affected by QUE and DAY × QUE (*p* > 0.05). Likewise, the protein abundance of occludin, a major component of tight junction protein complex, was not altered by quercetin supplementation (QUE: *p* > 0.05; DAY × QUE: *p* > 0.05). It must also be noted that the distal jejunal FD4 flux was not affected by DAY (*p* > 0.05), potentially signifying that the distal jejunal barrier function was not compromised during the weaning transition. Still, the distal jejunal occludin protein abundance was two-fold higher on d5 versus d14 post-weaning (*p* ≤ 0.05).

### 3.5. Principal Component Analysis

**Figure 5:**
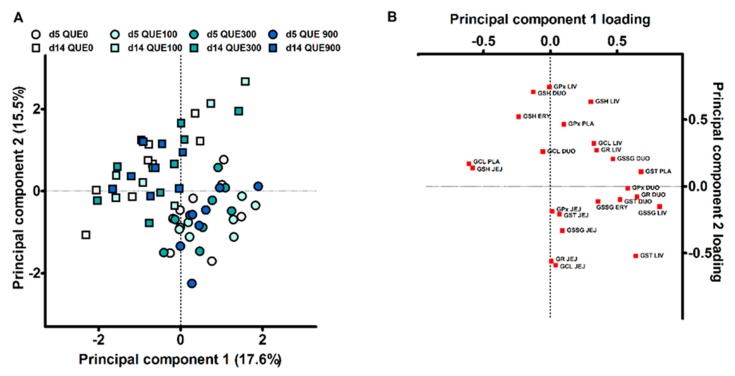
Principal component analysis of glutathione redox system in four different tissues of piglets at d5 and d14 post-weaning (DAY), fed a diet containing either 0, 100, 300, or 900 mg quercetin/kg diet (QUE) during the first 14d post-weaning: (**A**) Scores plot representing the 64 individual piglets in the multivariate space of the first two principal components. Animals can be visually clustered according to day post-weaning (d5 or d14). (**B**) Loading plot of the two major principal components. GPx = glutathione peroxidase, GR = glutathione reductase, GST = glutathione transferase; GCL = glutamate-cysteine ligase; GSH = glutathione; GSSG = glutathione dissulphide; PLA = plasma; ERY = erythrocyte; DUO = duodenum; JEJ = distal jejunum; LIV = liver.

A first PCA was executed, including all measurements on the GSH redox system at d5 and d14 post-weaning (23 variables), in order to evaluate if an overall response of dietary quercetin could be deduced. The first two principal components (PC) explained 17.6 and 15.5% of the variance. The subject score plot for PC1 vs. PC2 is shown in Figure 5A, and component loadings are presented in Figure 5B. Here, it seems that the objects did not cluster according to dietary treatment on d5 nor d14 post-weaning. This was statistically confirmed, as QUE and DAY × QUE did not affect the scores for PC1 and PC2 (*p* > 0.05). In contrast, subjects clearly clustered according to the day post-weaning. DAY had a significant effect on the scores for PC1 and PC2 (*p* < 0.001). The highest loadings in PC1 were the hepatic GSSG level (r = 0.818), the plasma GST activity (r = 0.679), the duodenal GR activity (r = 0.647) and hepatic GST activity (r = 0679). Piglets on d5 post-weaning were thus characterized by higher hepatic GSSG levels, and increased activities of plasma and liver GST and duodenal GR. The most important loadings in PC2 were the hepatic GPx activity (r = 0.743), the duodenal GSH level (r = 0.707) and the hepatic GSH level (r = 0.634). The decreased levels of duodenal and hepatic GSH, and the lower GPx activity in the liver, are indicative for piglets at d5 versus d14 post-weaning.

**Figure 6:**
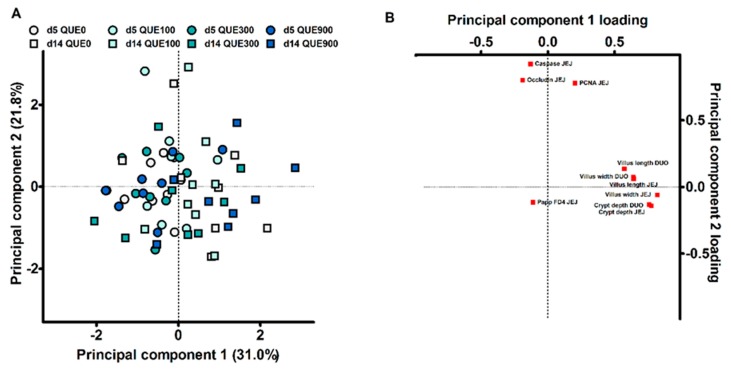
Principal component analysis of gut health parameters in the duodenum and distal jejunum of piglets at d5 and d14 post-weaning (DAY), fed a diet containing either 0, 100, 300, or 900 mg quercetin/kg diet (QUE) during the first 14d post-weaning: (**A**) Scores plot representing the 64 individual piglets in the multivariate space of the first two principal components. Animals can be visually clustered according to day post-weaning (d5 or d14). (**B**) Loading plot of the two major principal components. DUO = duodenum; JEJ = distal jejunum; PCNA = proliferating cell nuclear antigen; Papp = apparent permeability coefficient; FD4 = fluorescein isothiocyanate dextran.

The data on small intestinal histomorphology, mucosal protein abundance and barrier function were subjected to a second, separate PCA analysis (10 variables; results not shown). The first two principal components explained 51.8% of the variance (Figure 6A). PC1 predominantly contained high positive loadings for histomorphological characteristics, while PC2 was dominated by high positive loadings for the distal jejunal protein abundance of PCNA, Caspase-3 and occludin (Figure 6B). The distal jejunal FD4 flux received a relative low loading in both PC1 and PC2 (r < 0.200). Also here, subjects did not cluster according to dietary treatment, and neither QUE nor DAY × QUE influenced the scores for PC1 and PC2 (p > 0.05). Subjects did however visually cluster according to the day post-weaning. Only PC1 was significantly affected by DAY (p < 0.001). Consequently, d5 and d14 post-weaning could be clearly differentiated from each other based on histomorphological characteristics of the small intestine, rather than the barrier function or mitotic/apoptotic protein abundance.

## 4. Discussion

### 4.1. Quercetin Supplementation Promoted the Hepatic GST Activity in the Occurrence of Oxidative Stress

Effects of quercetin on the GSH redox status have been most often studied in experimental models for colitis in rats. Several of these studies report increased colonic mucosal GSH levels when quercetin or its glycosides are orally provided [29,30,54]. This effect, however, seems to depend on the dose of quercetin. Sánchez de Medina et al. (1996) [28] for example applied doses of 0.125, 0.25, 0.5, 1, 5, 10, 25 and 50 mg quercetin per kg body weight, and found that only 5 and 10 mg/kg increased GSH in the colon. A dose-response study by Cruz et al. (1997) [27], although using rutin and not quercetin, found quite similar doses to be effective. Doses of 5 to 25 mg/kg body weight were effective in increasing colonic GSH levels. Levels of 25 mg quercitrin/kg were also found to be effective after three days of oral administration in rats that were not subjected to an experimental model of colitis, but GSH values normalized four days later [55]. This study also reported increased GSH levels in the ileal mucosa, whereas jejunal concentrations remained unaffected. Meyers et al. (2008) [56] further demonstrated that also hepatic GSH can be altered by quercetin administration in unchallenged mice, and this was shown to depend on the food source or glycosidic moiety linked to the quercetin molecule. Dietary quercetin but not rutin or quercetin glycosides from onion and apple peels, applied at an equal dose of 200 mg quercetin per kg diet, increased hepatic GSH levels after seven days of administration. In the current study, doses of 100 to 900 mg quercetin per kg diet, equal to 2.5–25 mg quercetin/kg body weight, were applied in pigs. The hepatic GSH and GSSG levels, and consequently the GSH/GSSG E_h_, were however not altered by quercetin supplementation.

It was remarkable that the hepatic GST activity increased at d5 post-weaning when quercetin was added to the diet. This could relate to the phase II metabolism of quercetin by GST in the liver, where glutathionylation of quercetin takes place in order to promote its excretion [57]. Several glutathionylated quercetin metabolites have already been identified in murine hepatic suspensions and in human urine [58]. Quercetin yet only increased the hepatic GST activity at d5 post-weaning. Values on d14 post-weaning were low and were not affected by quercetin supplementation. The previously cited authors [58] also mention that conjugation reactions between quercetin and GSH occur spontaneously and are not accelerated by GST (unpublished results). Even more, some in vitro work demonstrated that quinone-type oxidation products of quercetin can make covalent modifications to certain cysteine residues of GST, and thereby inhibit its activity. These modifications were also found to be reversible, and for instance, ascorbic acid and GSH could prevent this inhibition [12]. Connected to this, one in vivo study reported decreased hepatic GST activities when 2000 mg quercetin per kg diet was fed to rats for three weeks [11]. This points out that, although quercetin is known as a powerful antioxidant, certain conditions can lead to a pro-oxidant behavior of quercetin and its derivates. This behavior was particularly prompted in a Fenton-like reaction system [59].

The pro-oxidant nature of quercetin has also been linked to the ability to upregulate GSH synthesis by altering GCL activity [9,10]. The exact mode of action still is not fully elucidated, but it was proposed to be associated with the feedback regulation of GCL when oxidized quercetin radicals cause GSH depletion [60,61]. In our study, we did not observe evidence for this. GCL activities remained unaffected in all measured tissues. Distal jejunal GSH levels were decreased when 300 mg quercetin/kg diet was supplied, but not at doses of 100 or 900 mg/kg. The unaltered MDA levels and further lack of treatment effect on the GSH redox system do not support that quercetin exhibited such an effect in the current experiment. An alternative mode of action depicts that oxidized quercetin radicals can react with thiols of important cell signaling proteins such as Kelch-like ECH-associated protein 1 (Keap1). The redox status of Keap1 affects its binding affinity for Nuclear factor erythroid 2–related factor 2 (Nrf2). The subsequent release and translocation of Nrf2 to the nucleus leads to the transcriptional upregulation of the antioxidant response [9,10]. In our study, we did not detect upregulated GPx, GR or GCL activities by quercetin, nor did the PCA analysis showed an overall change in the GSH redox system when quercetin was supplemented. We only observed a specific change in the hepatic GST activity on d5 post-weaning when quercetin was supplemented. This was recently also observed by Gao et al. (2017), when feeding a particularly high dose of 5000 mg/kg diet to rats for 6 weeks (1665) [13]. Transcriptional upregulation of GST via the Keap1/Nrf2 signaling pathway is known to take place in an oxidative stress event [62,63], which supports our observations in the liver. The hepatic GSSG level was increased and consequently, the GSH redox status was more oxidized on d5 post-weaning, and the GST activity was increased in the control treatment on d5 versus d14 post- weaning. Quercetin thus further promoted GST upregulation in an oxidative stress event. This points out that the altered hepatic GST activity was not destined to facilitate quercetin elimination but intended to empower other functions of GST. For example, protein glutathionylation by GST could have assisted in post-translational protein regulation and cell signaling [64,65,66], or in the protection of critical protein thiols from irreversible oxidation [67]. The presence of protein-GSH mixed disulphides and the glutathionylation of specific target proteins were not investigated in our study and would have been valuable to confirm this statement. Earlier research did for instance already report altered protein glutathionylation profiles in mice when quercetin was added to the diet [56]. GST further could have been involved in the removal of xenobiotics such as lipid peroxidation products [68], but this could not be confirmed by our measurements. MDA values were not affected by quercetin throughout the experiment. Quercetin-rich onions however did demonstrate to influence plasma lipid profile of pigs when fed a high-fat diet [69,70], which could relate to lower circulating levels of MDA-modified low density lipoprotein, and thus a reduced risk for coronary heart disease [71]. With regard to the small intestine, although we observed a more oxidized GSH redox pool and an increased distal jejunal GST activity on d5 post-weaning, quercetin did not further upregulate small intestinal GST as observed in the liver.

### 4.2. Quercetin Supplementation Did not Improve the Small Intestinal Functionality

Numerous in vivo studies, almost exclusively in rodents, have tested the health benefits of flavonols in pro-inflammatory diseases such as chronic inflammatory bowel disease [2,3]. These insights are valuable since weaned piglets endure a pathology that, to a certain extent, parallels with a condition of inflammatory bowel disease. For instance, newly weaned piglets typically display poor growth or even weight loss during the first five days post-weaning [72], and this is often followed by a surge of diarrhea [73]. This was also observed in the current study and aligns with the outcome of several different experimental models for colitis in rodents [28,29,36,74,75]. Importantly, Galvez et al. (1993a; 1993b; 1995) demonstrated that dietary quercetin and quercitrin at doses of 25 to 50 mg/kg body weight could reduce diarrhea prevalence in challenged rodents [32,33,34]. Further investigation indicated that the effects of quercitrin were attributed to quercetin release generated after glycoside cleavage by the intestinal microbiota [76]. These doses of quercetin were quite similar to the dose range in the current study, where quercetin was supplemented at 100 to 900 mg/kg diet (2.5–25 mg/kg body weight). However, we did not observe a similar effect during the weaning transition of piglets. This could be due to the different etiology, whereas in piglets, diarrhea is typically associated with malabsorption [77] and the proliferation of enterotoxigenic *Escherichia coli* (ETEC) in the intestine [78].

Besides diarrhea, experimentally induced colitis in rodents also results in epithelial degeneration in the colon [74,75,79]. Similar in the pig, weaning is followed by instantaneous villus atrophy and crypt hyperplasia in the small intestine. In both cases this indicates an increased rate of cell loss and/or a reduced rate of cell renewal [80]. In the current study, weaning-induced villus atrophy most probably did occur [81], but this could not be confirmed by comparing d5 with d14 post-weaning. The deeper crypts at both intestinal sites and the higher protein abundance of PCNA and caspase-3 in the distal jejunum at d5 post-weaning do substantiate that a process of damage and recovery took place in the small intestine upon weaning. Quercetin supplementation, yet, did not impact on the histological characteristics of the small intestine, and neither did it influence the PCNA or caspase-3 protein abundance in the distal jejunum. The PCA analysis could further only differentiate the subjects according to the day post-weaning. This indicates that the beneficial effects of quercetin in the colon [24,26,29,30,31,35,62,82] and the small intestine [62], observed in experimentally challenged rodents, are not easily translated to the weaner pig. One study in pigs did observe an increased jejunal villus length by dietary quercetin supplementation (25 mg/kg diet) prior to the induction of transport stress, and also reported altered jejunal tight junction mRNA expression [22]. Indeed, quercetin is also considered to modulate the tight junction distribution, either via directly interacting with intracellular signaling molecules such as protein kinases [83,84], or through the GSH-modulating properties of quercetin and its consequences on multiple facets of tight junction regulation [15]. In our study, the distal jejunal barrier function did not differ between the dietary treatments. Likewise, the small intestinal GSH redox system was not affected by quercetin, not even in the event of oxidative stress at d5 post-weaning. This suggests that redox regulation of cell cycling [17,18] and tight junction distribution [15,83,84] was not affected by quercetin in our trial.

## 5. Conclusions

The data presented in this study demonstrate that quercetin, supplemented at 100, 300 and 900 mg/kg diet, upregulated the hepatic GST activity at d5 post-weaning, when piglets experienced redox disturbance in the liver and small intestinal mucosa. This potentially amplified protein glutathionylation or xenobiotic conjugation, which are important instruments in the response to redox disturbance. This effect was however not observed in the small intestinal mucosa. Quercetin also did not prevent intestinal damage upon weaning, nor did it support intestinal recovery until d14 post-weaning. As a result, quercetin supplementation did not improve animal performance or health during the weaning transition.

## Figures and Tables

**Table 1:** Ingredient and calculated nutrient composition of the weaner and starter diet, fed to piglets from d0–14 and d14–42 post-weaning, respectively.

Ingredient Composition [%]	Weaner Diet	Starter Diet
Barley	25.00	35.00
Wheat	23.38	15.00
Toasted soybeans	19.97	4.00
Corn	15.00	17.31
Sweet whey powder	5.00	
Sugar beet pulp	2.00	2.00
Lard		2.00
Lactic acid (52%)	1.92	
Soybean meal (49% crude protein)	1.53	19.34
Lactose	1.27	
Potato protein	1.22	
Monocalciumphosphate	0.79	1.15
Limestone (fine) 38% Ca	0.58	1.00
Soybean oil		0.73
Salt	0.28	0.45
L-lysine HCl	0.48	0.45
DL-methionine	0.25	0.21
L-threonine	0.22	0.19
L-valine	0.22	0.12
L-tryptophan	0.07	0.05
Vitamin and mineral premix ^1^	1.00	1.00
**Calculated Nutrient Levels**		
Net energy ^2^ [MJ/kg]	10.1	9.8
Crude protein [g/kg]	169	180
Ether extract [g/kg]	54.9	53.0
Digestible lysine ^2^ [g/kg]	11.5	11.0
Digestible methionine + cysteine ^2^ [g/kg]	6.9	6.6
Digestible threonine ^2^ [g/kg]	7.1	6.8
Digestible tryptophan ^2^ [g/kg]	2.3	2.2
Digestible valine ^2^ [g/kg]	8.1	7.7

^1^ The mineral and vitamin premix supplied as the following (per kg diet): Vitamin A, 10,000 IU; Vitamin D3, 2000 IU; Vitamin E; 54 IE Vitamin K3, 1 mg; Vitamin B1, 1.2 mg; Vitamin B2, 3.7 mg; Vitamin B3, 12 mg; Vitamin B5, 25 mg; Vitamin B6, 2 mg; Vitamin B12, 0.030 mg; Biotin, 0.2 mg; Niacine, 20 mg; Folic acid, 0.8 mg; Fe^2+^, 100 mg Zn^2+^, 100 mg; Cu^2+^, 15 mg; Mn^2+^, 80 mg; Se^6+^, 0.3 mg; I, 1 mg, BHT, 75 mg/kg; ethoxyquin, 37.7 mg/kg; choline, 500 mg; phytase, 500 FTU. ^2^ Net energy for pigs CVB (1997), Centraal Veevoederbureau, Lelystad, The Nederlands [42].

**Table 2:** The effect of quercetin at doses of 0, 100, 300, or 900 mg/kg diet (QUE) fed during the first 14 days post-weaning on animal performance of piglets from d0 to d42 post-weaning (*n* = 8).

	QUE				SEM	*p*-Value
	0	100	300	900		
Body weight (BW) [kg]						
d0	5.88	5.88	5.88	5.88	0.03	0.956
d5	6.00	5.99	5.82	5.96	0.04	0.174
d14	6.87	6.73	6.54	7.06	0.11	0.201
d28	11.63 ^ab^	11.46 ^ab^	11.22 ^a^	12.18 ^b^	0.15	0.039
d42	18.06 ^ab^	17.92 ^ab^	17.70 ^a^	19.09 ^b^	0.21	0.029
Average daily gain (ADG) [g/d]						
d0–5	24	19	-7	16	7	0.256
d5–14	91	81	80	113	10	0.376
d14–28	310	310	306	337	5	0.056
d28–42	478	482	487	523	8	0.067
d0–42	286 ^ab^	284 ^ab^	282 ^a^	313 ^b^	5	0.030
Average daily feed intake (ADFI) [g/d]						
d0–5	71	67	52	63	5	0.348
d5–14	178	168	156	197	9	0.183
d14–28	436	432	421	469	9	0.120
d28–42	740 ^a^	747 ^ab^	759 ^ab^	807 ^b^	11	0.046
d0–42	396	395	389	429	7	0.070
Feed conversion ratio (FCR) [ADG/ADFI]						
d0–5	0.308	0.206	−0.912	0.237	0.333	0.346
d5–14	0.489	0.473	0.276	0.575	0.102	0.598
d14–28	0.712	0.718	0.733	0.721	0.010	0.848
d28–42	0.646	0.644	0.642	0.648	0.004	0.892
d0–42	0.722	0.720	0.728	0.734	0.006	0.736

^a,b^ Within a row, least square means without a common superscript letter differ significantly (*p* ≤ 0.05).

**Table 3:** The effect of quercetin at a dose of either 0, 100, 300, or 900 mg/kg diet (QUE) fed during the first 14d post-weaning on the malondialdehyde (MDA) concentration in different tissues of piglets at d5 and d14 post-weaning (DAY) (*n* = 8).

DAY	5				14				SEM	*p*-Value		
QUE	0	100	300	900	0	100	300	900		DAY	QUE	DAY × QUE
MDA [nmol/g prot.]												
Plasma^1^	9.01	8.46	8.62	7.94	7.00	7.55	7.28	7.38	0.12	<0.001	0.694	0.202
Duodenal mucosa	361	453	398	383	310	347	304	284	12	<0.001	0.124	0.909
Distal jejunal mucosa	483	556	491	519	472	403	472	419	17	0.042	0.994	0.398
Liver	1380	1057	1155	1149	875	974	982	948	32	<0.001	0.663	0.125

^1^ Expressed in nmol/mL.

**Table 4:** Significance levels for treatment effects on the glutathione redox system in blood of piglets at d5 and d14post-weaning (DAY), fed a diet containing 0, 100, 300, or 900 mg quercetin/kg diet (QUE) during the first 14d post-weaning (*n* = 8).

*p*-Value	GPx	GST	GCL	GSH	GSSG	GSH/GSSG E_h_
DAY	0.003	0.009	<0.001	0.001	0.039	<0.001
QUE	0.776	0.741	0.974	0.077	0.925	0.115
DAY × QUE	0.076	0.146	0.936	0.912	0.221	0.624

**Table 5:** Significance levels for treatment effects on the glutathione redox system in the duodenal mucosa of piglets at d5 and d14post-weaning (DAY), fed a diet containing either 0, 100, 300, or 900 mg quercetin/kg diet (QUE) during the first 14d post-weaning (*n* = 8).

*p*-Value	GPx	GR	GST	GCL	GSH	GSSG	GSH/GSSG E_h_
DAY	0.044	0.002	0.021	0.200	<0.001	0.542	<0.001
QUE	0.787	0.346	0.837	0.650	0.614	0.664	0.682
DAY × QUE	0.355	0.750	0.624	0.288	0.891	0.939	0.742

**Table 6:** Significance levels for treatment effects on the glutathione redox system in the distal jejunal mucosa of piglets at d5 and d14 post-weaning (DAY), fed a diet containing either 0, 100, 300, or 900 mg quercetin/kg diet (QUE) during the first 14d post-weaning (*n* = 8).

*p*-Value	GPx	GR	GST	GCL	GSH	GSSG	GSH/GSSG E_h_
DAY	0.976	0.009	0.367	0.002	< 0.001	0.021	< 0.001
QUE	0.373	0.101	0.694	0.176	0.050	0.567	0.455
DAY × QUE	0.561	0.492	0.347	0.480	0.644	0.979	0.853

**Table 7:** Significance levels for treatment effects on the glutathione redox system in lever tissue of piglets at d5 and d14post-weaning (DAY), fed a diet containing 0, 100, 300, or 900 mg quercetin/kg diet (QUE) during the first 14d post-weaning (*n* = 8).

*p*-Value.	GPx	GR	GST	GCL	GSH	GSSG	GSH/GSSG E_h_
DAY	< 0.001	0.297	< 0.001	0.546	0.258	< 0.001	< 0.001
QUE	0.775	0.309	0.018	0.905	0.584	0.485	0.428
DAY × QUE	0.519	0.463	0.036	0.745	0.864	0.864	0.988

**Table 8:** The effect of quercetin at doses of either 0, 100, 300, or 900 mg/kg diet (QUE) during the first 14d post-weaning on the small intestinal histomorphology, barrier function and tight junction and mitotic/apoptotic protein abundance in piglets at d5 and d14post-weaning (DAY) (*n* = 8).

DAY	5				14				SEM	*p*-Value		
QUE	0	100	300	900	0	100	300	900		DAY	QUE	DAY × QUE
Duodenum												
Villus length [µm]	255	257	264	254	243	239	246	300	7	0.461	0.477	0.361
Villus width [µm]	97	105	97	102	97	94	96	105	2	0.154	0.702	0.597
Crypt depth [µm]	215	271	199	253	210	249	206	267	4	< 0.001	0.424	0.791
Villus/crypt ratio [µm/µm]	1.19	0.94	1.34	1.01	1.17	1.00	1.20	1.13	0.03	0.001	0.492	0.456
Distal jejunum												
Villus length [µm]	220	252	268	224	177	224	198	262	9	0.180	0.350	0.177
Villus width [µm]	87	93	87	95	86	86	78	101	2	0.010	0.697	0.135
Crypt depth [µm]	159	211	164	203	154	206	256	219	4	< 0.001	0.911	0.832
Villus/crypt ratio [µm/µm]	1.39	1.21	1.62	1.12	1.16	1.10	1.27	1.21	0.05	0.046	0.409	0.384
PCNA [fmol/mg prot.]	3.23	4.08	3.26	4.57	3.22	4.12	3.12	4.37	0.19	0.007	0.956	0.966
Caspase-3 [fmol/mg prot.]	12.4	10.7	11.6	11.0	12.5	9.3	12.4	9.1	0.6	0.090	0.970	0.856
P_app_ FD4 [× 10^−7^ cm/s]	10.0	9.3	8.8	11.0	8.94	12.0	7.5	8.3	0.8	0.386	0.671	0.831
Occludin [fmol/mg prot.]	1.67	1.68	3.10	1.12	1.79	0.77	1.58	0.62	0.17	0.005	0.168	0.231

P_app_ = apparent permeability coefficient, FD4 = fluorescein isothiocyanate dextran, PCNA = proliferating cell nuclear antigen.
